# 三级淋巴结构在非小细胞肺癌预后及免疫治疗中的研究进展

**DOI:** 10.3779/j.issn.1009-3419.2023.101.23

**Published:** 2023-08-20

**Authors:** Ying LIU, Lei XIONG, Ruoxue CAI, Yue CHEN, Jinjun YE, Bo SHEN, Guoren ZHOU

**Affiliations:** ^1^210009 南京，南京医科大学附属肿瘤医院，江苏省肿瘤医院，江苏省肿瘤防治研究所; ^1^The Affiliated Cancer Hospital of Nanjing Medical University, Jiangsu Cancer Hospital, Jiangsu Institute of Cancer Research, Nanjing 210009, China; ^2^210002 南京，东部战区总医院; ^2^General Hospital of Eastern Theater Command, Nanjing 210002, China

**Keywords:** 三级淋巴结构, 肺肿瘤, 免疫治疗, Tertiary lymphoid structure, Lung neoplasms, Immunotherapy

## Abstract

肺癌是当前癌症死亡的首要原因，其中非小细胞肺癌（non-small cell lung cancer, NSCLC）占肺癌的85%。免疫治疗显著改善了NSCLC患者的临床预后，但肿瘤微环境具有复杂性和异质性，仍有部分患者不能受益于免疫治疗，因此有必要探索NSCLC免疫治疗的有效预测生物标志物。三级淋巴结构（tertiary lymphoid structure, TLS）是与次级淋巴器官（secondary lymphoid organs, SLO）高度相似的异位淋巴器官，有研究发现TLS的存在与多种实体瘤（包括NSCLC）免疫治疗的良好预后密切相关。本文对TLS在NSCLC预后及免疫治疗中的作用进行综述，为筛选NSCLC免疫治疗适宜人群及制定个性化、精准化治疗方案提供参考。

## Abstract (en)

Lung cancer is the leading cause of cancer death, and non-small cell lung cancer (NSCLC) accounts for 85%. Immunotherapy has significantly improved the clinical prognosis of patients with NSCLC. However, because of the complexity and heterogeneousness of the tumor microenvironment, only a subset of individuals can benefit from immunotherapy. Therefore, it is necessary to explore effective predictive biomarkers for immunotherapy of NSCLC. Tertiary lymphoid structure (TLS) is an ectopic lymphoid organ that is highly similar to secondary lymphoid organs (SLO), and the presence of TLS has been found to be closely associated with a good prognosis in immunotherapy for a variety of solid tumors, including NSCLC. This article provides a review of the prognostic role of tertiary lymphoid structures in immunotherapy of NSCLC, in order to offer references for screening suitable candidates for immunotherapy of NSCLC and develop personalized and precise treatment plans.

肺癌发病率在全球恶性肿瘤中位居第二^[1]^，其中非小细胞肺癌（non-small cell lung cancer, NSCLC）约占肺癌的85%。免疫检查点抑制剂的临床应用显著改善了NSCLC患者的临床预后。但是，肿瘤微环境具有复杂性和异质性，因此仍有部分患者不能受益于免疫治疗^[2]^。目前已知的预测因子包括肿瘤组织中程序性细胞死亡配体1（programmed cell death ligand 1, PD-L1）的表达^[3]^和肿瘤突变负荷（tumor mutational burden, TMB）^[4⇓-6]^等，评估肿瘤微环境中的详细成分（如骨髓细胞和其他免疫细胞）的研究正在不断开展^[7]^。三级淋巴结构（tertiary lymphoid structure, TLS）是与次级淋巴器官（secondary lymphoid organs, SLO）高度相似的异位淋巴器官，有研究^[8,9]^发现TLS的存在与多种实体瘤（包括NSCLC）免疫治疗的良好预后密切相关。本文对TLS在NSCLC免疫治疗中的预后价值进行综述，旨在为筛选NSCLC免疫治疗适宜人群以及开展后期研究提供参考。

## 1 TLS的基本介绍

### 1.1 TLS的组成

TLS通常在生理条件下不会形成，它是在慢性炎症部位非淋巴组织中发育的异位淋巴器官^[10]^，在各种病理生理学情况下发展，包括自身免疫性疾病^[11]^、感染性疾病^[12]^和肿瘤^[13]^等，驱动一系列环境依赖性效应。当前已经在多种实体瘤（包括黑色素瘤^[14]^、结直肠癌^[15]^、乳腺癌^[16]^和NSCLC^[17]^等）中发现，TLS与肿瘤免疫治疗的预后密切相关。TLS主要由B细胞、T细胞和树突状细胞（dentritic cell, DC）组成^[18]^。在肿瘤微环境中，TLS作为免疫细胞聚集体，尽管主要由内部CD20^+ ^B细胞区和周围CD3^+ ^T细胞区组成^[19]^，不同的DC也同时构成DC细胞群。TLS为DC将相邻肿瘤抗原局部呈递给T细胞，以及为B细胞、T细胞的活化和增殖提供场所，从而产生记忆性辅助T细胞、效应记忆性细胞毒性细胞、记忆性B细胞和产生抗体的浆细胞^[20,21]^。TLS的成熟度可分成三个阶段：早期TLSs（early-TLS, E-TLS）——无滤泡树突状细胞（follicle dendritic cell, FDC）的致密淋巴细胞聚集体；初级滤泡样TLSs（primary follicle-like TLS, PFL-TLS）——B细胞簇，无生发中心的FDC网；次级滤泡样TLS（secondary follicle-like TLS, SFL-TLS）——B细胞簇，具有生发中心的FDC网。

高内皮小静脉作为TLS的重要组成部分，起着招募免疫细胞的作用^[22]^。TLS的形成是一个多步骤的过程，这些免疫细胞、基质细胞和细胞外基质组分的逐渐积累和组织化最终形成功能性的淋巴组织，从而实现局部适应性免疫应答。因此，TLS的存在与良好的预后和对免疫治疗的反应有关^[23]^。

### 1.2 TLS的特点及临床应用

关于TLS在肿瘤中的分布位置尚无统一定论，这和它的位置难以确切评估相关。当前较多研究的是其在肿瘤周边和肿瘤内位置与预后的关系。尽管在肝细胞癌和胆管癌中都有研究^[24,25]^证实肿瘤内TLS相较于肿瘤周边与肿瘤更好的预后相关，但是大多数肿瘤中尚未发现TLS分布位置与预后的确切关系。此外，TLS在肿瘤中的成熟状态具有异质性，不同成熟状态的TLS在肿瘤中的预后作用可能不同。具有高成熟TLS的NSCLC患者免疫治疗预后更好^[26]^，这在食管癌^[27]^和胰腺癌^[28]^等多种实体瘤中都有证实。

由于TLS的位置、成熟度和丰度等均可能对肿瘤患者免疫治疗的预后有影响，因此研究者们综合TLS的特征，建立评分系统，以完善TLS在预后作用中的临床应用。根据TLS在肿瘤周围组织和肿瘤内的TLS丰度进行评分是应用较多的方法^[8]^，以此为基础的免疫分型系统在很大程度上提高了TLS预后预测潜力，有助于精准治疗的推动。总的来说，TLS的组成、位置和空间结构的异质性，在临床应用上体现在不同空间分布下其密度与肿瘤预后的相关性。评分过程中不可避免地会出现误差，这主要是由于不同病理学家的评估标准不同以及评估过程中出现的问题，未来需要对TLS各个方面进行细化，对评分标准进行更严格的规定，以提高其对肿瘤预后作用的预测精确性。

### 1.3 TLS的检测方法

对肿瘤组织切片进行苏木素-伊红（hematoxylin-eosin, HE）染色是检测TLS最简单的方法。目前，通过免疫组化在组织中检测TLS已成为较为普遍的形式，且特异性较好，可鉴定CD4^+ ^T细胞、CD8^+ ^T细胞和CD20^+ ^B细胞^[29]^。但TLS阳性的肿瘤组织中提取到的mRNA的多个特征比较具有异质性，且该方法无法探究TLS中免疫细胞互相作用的情况，因此仍需要开发更可靠的TLS检测方法。多重免疫荧光技术可以在组织切片上标记TLS相关细胞，进行识别和定量分析，区分不同亚型和成熟度的TLS^[30]^，是当前使用较多的检测方法，用于评估TLS成熟度、密度以及组成对肿瘤预后的影响。转录组测序技术是高通量分析的理想选择，但是受限于参考数据的偏差，并且需要大量样本才能建立参考数据并进行验证^[31,32]^。空间组学的进步使得TLS的检测方法过渡到数字空间分析技术和多重免疫荧光技术，可以从分子层面更清晰地探索TLS的组成和功能^[33]^。

## 2 TLS在NSCLC预后中的作用

既往有研究^[34]^证实，TLS评分是一个独立的阳性预后因素，提高了各病理分期的预后能力，因此有望完善可切除NSCLC的肿瘤原发灶-淋巴结-转移（tumor-node-metastasis, TNM）分期。一项纳入112例接受完全切除的IB期肺腺癌患者的研究^[35]^也证实了TLS作为独立预后因素的作用。该研究中，TLS阳性患者与TLS阴性患者相比，TLS阳性与更长的无复发生存期（recurrence free survival, RFS）相关（HR=0.47, 95%CI: 0.23-0.88, P=0.02），多因素分析发现TLS是RFS的独立预后因素，且TLS阳性和阴性患者的PD-L1表达（P=0.54）和TMB（P=0.39）均无明显差异。该研究中通过HE染色和免疫组化评估TLS是否存在，表明NSCLC中存在TLS是有利的预后因素，且与肺腺癌患者的PD-L1表达无关。在软组织肉瘤中也证实存在TLS患者的6个月无进展生存率（non-progression rate, NPR）和客观缓解率（objective response rate, ORR）分别为40%（95%CI: 22.7%-59.4%）和30%（95%CI: 14.7%-49.4%），远高于全队列的NPR和ORR^[36]^。一项纳入147例NSCLC患者的单中心回顾性研究^[37]^中，TLS高表达组的无病生存期（disease-free survival, DFS）高于低表达组（P=0.027），证实II、III期NSCLC患者手术后TLS高表达与预后良好相关。此外，该研究还发现外周血中低中性粒细胞淋巴细胞比值和少量抗原呈递细胞可表明肿瘤微环境中存在TLS。

TLS组成和空间结构决定其与免疫治疗的关系密不可分，因此它在化学治疗中的预测作用十分有限。一项纳入来自114例接受铂类化疗作为转移性一线治疗NSCLC患者的研究^[38]^中，TLS阳性组的ORR和中位无进展生存期（progression-free survival, PFS）与TLS阴性组之间无差异（50% vs 48.8%，P=1；4.7个月 vs 4.4个月，P=0.9），表明TLS不能预测接受化疗的转移性NSCLC患者的临床结果。然而，有研究^[39]^显示TLS与新辅助化疗可切除NSCLC患者的主要病理反应密切相关，亦有研究^[40,41]^报道新辅助化疗增加细胞毒性T细胞和B细胞浸润，并降低可切除NSCLC患者肿瘤中叉头框蛋白P3（forkheadbox P3, FoxP3）T细胞的密度。

总结来说，TLS是否存在、成熟度和表达度等都会对NSCLC患者的预后产生影响，并且主要是与接受免疫治疗的患者相关。但是TLS的特征中究竟何者是最佳选择尚无定论，因此当前越来越多研究基于TLS的分布位置，结合其丰度等特点，建立TLS评分系统，旨在提高其对NSCLC预后的预测能力。

## 3 TLS对NSCLC免疫治疗的影响

研究者们^[42]^通过建立小鼠模型研究TLS与肺腺癌免疫治疗反应之间的潜在机制，发现小鼠模型中有效的免疫疗法需要形成CXCL13依赖性TLS，并且TLS与肺腺癌对免疫治疗的反应相关。多项研究^[43,44]^都证明免疫治疗可以诱导肿瘤小鼠模型的TLS形成和成熟。在肺腺癌患者中，研究者们通过免疫组化研究TLS表型，将TLS分为高成熟组和低成熟组，证实成熟TLS与引流淋巴结中细胞毒性淋巴细胞数量增加和淋巴细胞转移频率降低相关，这表明成熟的TLS 可能通过活化淋巴细胞增强抗肿瘤免疫治疗效果^[45]^。在食管癌^[46]^、胆管癌^[25]^和结直肠癌^[15]^等实体瘤中，也证实了TLS的空间分布和丰度与免疫治疗反应显著相关。

新辅助治疗可降低原发灶肿瘤负荷，为患者争取手术机会，随着精准治疗理念的深入，新辅助治疗方案也从既往的新辅助化疗发展至新辅助靶向治疗、新辅助免疫治疗和新辅助内分泌治疗等多种策略。辅助免疫治疗可以诱导机体产生持久的抗肿瘤免疫反应，并清除微小残留病灶。因此，可切除肺癌患者可通过新辅助免疫治疗方案获得更低的术后复发或远处转移风险，从而有更好的临床获益^[47⇓-49]^。探索新辅助免疫治疗的适宜人群也刻不容缓。截至目前，TLS在肺癌免疫治疗预后中的价值已被大量研究证实。越来越多的研究者们开始探索TLS在肺癌新辅助免疫治疗中的预后作用。一项回顾性研究^[50]^探讨了肿瘤微环境与不同新辅助方式治疗的病理反应在NSCLC的相关性，研究中达到主要病理缓解（major pathological response, MPR）的患者有更高的TLS，达到完全病理缓解（pathologic complete response, PCR）的患者有显著较低的FoxP3 T细胞密度，从肿瘤微环境角度为理解新辅助免疫联合化疗优于单独化疗的机制提供新见解。TLS与病理反应的关系不仅存在于NSCLC中，在接受新辅助免疫联合化疗的乳腺癌患者中，TLS状态与PCR显著相关，TLS丰度对人表皮生长因子受体2（human epidermal growth factor receptor 2, HER2）阴性乳腺癌病理反应具有独立预测价值^[16]^。接受了伊匹木单抗和纳武利尤单抗双免治疗的尿路上皮癌患者中，达到完全缓解的患者可观察到TLS富集^[51]^。为探索新辅助免疫联合化疗方案的潜在分子机制，研究者们选取了IIIA期行手术切除的NSCLC患者，对其CD45免疫细胞行单细胞转录组和T细胞受体测序，这些患者都接受了新辅助免疫联合化疗，研究发现在肿瘤病变中，CXCL13和CXCL12趋化因子显著富集，证实新辅助免疫联合化疗刺激了TLS形成^[17]^。一项探索三级淋巴结结构的成熟度和丰度与接受新辅助免疫治疗的肺癌患者的疗效相关性的研究^[26]^发现，TLS高成熟度和高丰度组比低成熟度和低丰度组具有更好的DFS，多变量分析显示TLS成熟度在新辅助免疫联合化疗组中（P=0.014, HR=4.527, 95%CI: 1.351-15.167）是DFS的独立预测因子。该研究通过HE和免疫组化对TLS进行鉴定和定量分析，证实TLS成熟度与MPR相关，是接受新辅助免疫联合化疗的NSCLC患者DFS的独立预测因子，并且诱导TLS成熟可能是新辅助化疗免疫治疗在可切除NSCLC中的潜在作用机制。鉴于TLS不同特征在NSCLC预后和免疫治疗中的作用，本文在表1对相关研究进行了总结。

**表1:** TLS与NSCLC患者预后的相关性研究

Year	Detection of TLSs	Treatment strategy	*n*	Parameter of TLSs	Conclusions	Ref.
2021	Immunohistochemistry	NA	490	TLS score	The TLS score has the potential to improve TNM staging after NSCLC resection	^[34]^
2022	HE and immunohistochemistry	NA	112	The presence of TLS	Presence of TLS in NSCLC is a favourable prognostic factor	^[35]^
2022	Immunohistochemistry	NA	147	TLS expression	High TLS expression after surgery in patients with stage II and III NSCLC is associated with a good prognosis	^[37]^
2022	NA	Immunotherapy vs Chemotherapy	114	The presence of mature TLS	Mature TLS is a specific biomarker for immunotherapy and does not predict the outcome of chemotherapy in NSCLC	^[38]^
2022	Multiple immunofluorescence staining	Neoadjuvant chemoimmunotherapy vs Neoadjuvant chemotherapy	55	TLS density	Neoadjuvant chemoimmunotherapy is associated with increased CD8^+^ T -cell infiltration compared to chemotherapy alone, providing new insights into the mechanisms of neoadjuvant chemoimmunotherapy	^[50]^
2022	RNA sequencing	Neoadjuvant chemoimmunotherapy	48	Immunophenotype	Neoadjuvant chemoimmunotherapy stimulated TLS formation	^[17]^
2022	HE staining and immunohistochemistry	Naive vs Neoadjuvant chemotherapy vs Neoadjuvant chemoimmunotherapy	121	TLS maturation and abundance	TLS maturity is an independent predictor of DFS in NSCLC receiving neoadjuvant chemoimmunotherapy	^[26]^

TLS: tertiary lymphoid structure; NSCLC: non-small cell lung cancer; NA: not available; HE: hematoxylin-eosin; RNA: ribonucleic acid; DFS: disease-free survival; TNM: tumor-node-metastasis.

## 4 展望

在肿瘤微环境的背景下，TLS的形成和发展有利于调节抗肿瘤免疫应答并改善肿瘤患者对免疫检查点抑制剂的耐药性。当前多项研究探索了TLS在NSCLC免疫治疗中的预后作用，尤其在新辅助免疫疗法推进的同时，起着不可或缺的作用，但其具体作用机制仍不清楚。并且，由于TLS的检测方法多样，最常用的HE染色难以清晰识别TLS并进行评估，多重免疫荧光和转录组测序等尚不能在临床普遍应用，因此TLS在临床上的扩展受限。TLS的空间分布和丰度等都与NSCLC免疫治疗的预后相关，未来需进一步细化TLS结构和功能，综合TLS多种特征，建立完善的评分机制。并且后续在研究TLS作用机制的同时，探索TLS诱导、化疗药物和免疫治疗间的潜在协同作用将有利于制定更有效的治疗方案。未来在肿瘤微环境的推动下，肿瘤预后生物标志物的研究将越来越精准化，逐步实现NSCLC的个性化治疗。
